# A Prospective Multicenter Randomized Study to Assess the Impact of a Novel Catheter Coating on Clinical Bacteriuria

**DOI:** 10.3390/antibiotics15040369

**Published:** 2026-04-03

**Authors:** Mark Rochester, Catherine Rennie, Clare Hayes, Jean O’Driscoll, Maurizio Belci

**Affiliations:** 1 Norfolk & Norwich University Hospital, Norwich NR4 7UY, UK; 2 Stoke Mandeville Hospital, Aylesbury HP21 8AL, UK

**Keywords:** UTI antibiotics, urethral catheter, polymer coated catheter, urinary tract infections, catheter associated urinary tract infections, bacterial colonization

## Abstract

**Background:** Catheter-associated urinary tract infections (CAUTIs) are a common source of morbidity and antibiotic use. Camstent Ltd. surface coated catheters aim to reduce bacterial colonization and infection. This study compares outcomes with Camstent Coated Catheters (CCC) versus standard uncoated catheters, Standard Care (SC). Objectives: The aim of the study was to investigate the reduction in bacteriuria in CCC versus SC, uncoated catheters. **Methods:** This is a prospective, UK, multi-center, randomized study including an Intention to Treat (ITT) population and a Per Protocol (PP) population of 200 and 188 subjects respectively. The PP population was sub-divided into primary and secondary cohorts with 107 and 81 subjects considered for study randomization receiving either a CCC or SC respectively. The primary endpoints including time to infection, number of days of infection and incidence of infection, and secondary endpoints including time to symptoms, UTI antibiotic use, patient reported outcomes like patient discomfort and catheter blockage were evaluated at days 0, 3, 5, 7, 10, 14, 21 and 28. For statistical analysis, Hodges–Lehmann and Fisher’s exact test were used. **Results:** The primary end points: Colonization-free rates at day 7 were higher in the CCC group than in the SC group (79% versus 46%) and the difference persisted on day 14 (69% versus 39%) (*p* = 0.016); the mean number of days infected during the first 14 days was lower in the CCC group than the SC group (3 versus 4.6 days) (two-sided; *p* = 0.0117); the infection rates at day 14 were lower in the CCC group than SC group (33% versus 50%). This trend continued at days 21 and 28, with consistently lower infection rates in the CCC group than SC group but did not reach statistical significance (*p* > 0.05). The secondary endpoint is in the secondary cohort: Time to the development of symptoms defined as a UTI requiring antibiotics showed that zero cases occurred in the CCC compared with the SC group (0% versus 20%) at *p* = 0.0054; median time to symptoms in the SC group was 9.0 days; and expanding this endpoint to include the primary cohort revealed that symptomatic infections occurred at 4% in the CCC group and 20% in the SC group (*p* = 0.0007) with a longer median time to symptoms in the CCC group than SC (13.5 versus 7 days); UTI antibiotic use was significantly lower in the CCC group than the SC group (4% vs. 21%). **Conclusions:** Compared with SC, CCCs were associated with substantial reductions in bacterial colonization, symptomatic CAUTI, and antibiotic use, supporting their adoption within strategies to prevent CAUTIs and promote antimicrobial stewardship. Trial registration: This study was registered prospectively in the Clinical Trials Registry (NCT04461262; Study Details|NCT04461262|The Impact Of A Catheter Coating On Clinical Bacteriuria|r).

## 1. Introduction

Urinary tract infections (UTIs) are among the most common infectious diseases worldwide, with acute cystitis representing the most frequent clinical presentation, typically characterized by urgency, frequency, dysuria, and pyuria [1,2,3,4]. UTIs also pose a substantial burden in hospitalized patients, particularly older adults and individuals with comorbidities such as diabetes, malignancy, or those requiring indwelling urinary catheters (IUCs) [4]. CAUTI is a symptomatic infection of the bladder or kidneys in a person who is catheterized or has had a urinary catheter in place within the previous 48 h. This means the person must have signs or symptoms that are consistent with a urinary tract infection and a urinary catheter in place (or recently removed), and there is not another clear source of infection. Catheter-associated infections may originate from multiple points along the drainage system. As highlighted by Maki and Tambyah, microbial entry can occur at the urethral meatus, at junctions between the catheter and the urine collection system, and via the contamination of the urine collection bag, particularly during emptying [5]. Coated catheters will mostly decrease the meatus urethrae, but not the junctions between the catheter and urine collection bag, and therefore we would never see the complete absence of UTIs using coated catheters.

Globally, UTIs remain one of the most frequently reported healthcare-associated infections, with urinary catheterization identified as the principal modifiable risk factor [6,7,8]. During hospitalization, approximately 10–25% of patients received an IUC, around 20% of these patients developed CAUTIs [9], and patients with IUC also showed catheter blockages due to the deposition of debris/crystal formation incorporated into the biofilm of the catheter, leading to bladder stones and resulting in encrustation and catheter blockage causing significant patient pain and discomfort [10,11,12,13,14,15]. In a study, patients with frequent catheter blockage showed significantly higher urine pH, ammonium, and calcium concentrations. The most important causes for catheter blockage are bacterial urease activity and urine calcium content [10]. Approximately 36% of patients with IUC develop bladder stones within 8 years [11]. Failure to detect bladder stones may lead to catheter blockage [16]. In a prospective cohort study, Linsenmeyer et al. determined the accuracy of bladder stone detection via cystoscopy based on catheter encrustation in asymptomatic individuals with spinal cord injury, which showed in 85% of cases a positive result for catheter encrustation was associated with bladder stones [16].

In European acute-care settings, UTIs account for approximately 19% of healthcare-associated infections, with around two-thirds linked to IUC. A six-year study from a tertiary hospital in Greece reported a CAUTI rate of 5.28 episodes per 1000 catheter-days, highlighting the persistent clinical and epidemiological burden of CAUTIs [17]. In the UK, over 90,000 adults have IUC, and nearly one quarter (~24%) developed symptoms of CAUTI, emphasizing the significant burden among long-term catheter users [18]. CAUTIs showed an estimated annual economic burden costing $1.7 billion a year in the United States alone [6]. Against this backdrop, the present study evaluates the performance of CCC compared with SC in hospitalized patients requiring catheterization.

Catheterization compromises host defenses and facilitates microbial entry, resulting in urethral trauma, bacteriuria, and subsequent infection [4,17]. The daily risk of acquiring bacteriuria ranges from 3 to 7%, and nearly all patients develop bacteriuria after 30 days of catheterization [7,19]. Long-term catheter use (≥28 days) in particular is common among older adults and is associated with UTI rates of up to 50%, while additional complications such as mineral encrustation further challenge catheter maintenance [20,21].

The pathogenesis of CAUTIs is closely linked to biofilm formation on catheter surfaces, primarily mediated by uropathogens such as *Escherichia coli* [9,22]. Biofilms composed of extracellular polymeric substances shield bacteria from host immunity and impede antimicrobial penetration, leading to persistent infections [22]. To mitigate these risks, multiple catheter surface modifications have been developed, focused on discovering biomaterials to inhibit microbial adhesion and biofilm development. Catheters coated with antiseptic or antimicrobial compounds—including silver ions, antibiotics, and noble metal alloys (NMAs)—have demonstrated reductions in bacterial colonization [23,24,25,26,27,28,29]. Clinical studies evaluating antimicrobial catheter coatings have reported heterogeneous results. A Cochrane review limited to short-term catheterization (≤14 days) found no significant reduction in symptomatic CAUTI with silver-alloy-coated catheters compared with standard catheters [30]. More recent pooled evidence suggests that antimicrobial-coated catheters may reduce CAUTI risk in patients requiring longer-term catheterization (>14 days), supporting the evaluation of catheter performance over durations of up to 28 days [31]. These variable findings reflect differences in catheterization duration, patient populations, device materials, and definitions of infection. Silver alloy and hydrogel-coated devices such as Bactiguard^®^ catheters use noble metal alloy coatings to provide anti-infective and anti-biofilm properties [32] yet sustained clinical effectiveness across diverse settings remains uncertain.

Next-generation non-biocidal surface technologies have been introduced to address these limitations. The LubriShield™ coating utilizes a permanent superhydrophilic surface functionalized with a proprietary anti-fouling ligand, suppressing biofilm formation without continuous antimicrobial release. In vitro studies have demonstrated reductions in biofilm formation by up to 83%, and increased antibiotic susceptibility of adherent microorganisms by 78%, with activity maintained for up to 14 days [22]. However, these findings are based on preclinical models, and clinical evidence demonstrating reductions in CAUTI incidence in actual patients is not yet available. Similarly, Camstent catheters incorporate inert “non-stick” acrylate polymer coatings using dip coating technology that generate bacteriophobic surfaces; laboratory evaluations have shown ≥90% reductions in bacterial colonization using the copolymerization of ethylene glycol dicyclopentenenyl ether acrylate (referred to as monomer 4 in ref. [33]) with diethylene glycol methyl ether methacrylate (M4D), without bacterial lysis [33,34]. Unlike traditional antimicrobial-impregnated materials, the acrylate coating used to coat Camstent coated catheters contains no leachable agents and does not exert bactericidal pressure. Instead, its mechanism is purely physicochemical: it exploits the intrinsic properties of the polymer to arrest the transition from reversible to irreversible bacterial attachment [25,35]. This stable, biocompatible coating effectively prevents biofilm maturation while maintaining the mechanical flexibility and lubricity required for silicone Foley catheter applications. Recent clinical evidence indicates that catheter surface modifications may reduce biofilm-related complications. Kalenderski et al. reported that polymer-coated urinary catheters significantly decreased biofilm biomass, biomineral deposition, and fibrinogen surface conditioning compared with uncoated silicone catheters for up to 28 days, with no coating-related safety issues observed [36].

Despite these innovations, CAUTI risk remains strongly influenced by catheter management practices. Strategies such as minimizing inappropriate catheter use, ensuring early removal, and improving metal hygiene—particularly with chlorhexidine—have proven effective in reducing infection rates [37,38]. Duration of catheterization and catheter material type remain key determinants of CAUTI risk [39,40].

Given the clinical and economic burden of CAUTIs, the inconsistency of clinical evidence for currently available coated catheters, and the promising yet preclinical data supporting novel anti-fouling technologies, further evaluation of coating strategies is warranted. This prospective multicenter randomized study was therefore designed to assess the impact of a coated catheter on the incidence of clinical bacteriuria, specifically examining the number of days patients exhibit bacterial viable counts exceeding 10^5^ colony forming units (CFU)/mL when using the CCC compared with SC.

## 2. Results

The Intention To Treat (ITT) population and Per Protocol (PP) population were 200 and 188 subjects respectively. Within the PP population of 188 subjects, a number of subjects did not continue until day 28 for a selection of logistical reasons not associated with their catheterization. For these subjects, the last bacterial viable counts value was used for the statistical analysis of the remaining time points.

The ITT and PP populations were both sub-divided into primary and secondary endpoints. The non-infected subjects in National Health Service (NHS) hospitals were assigned to the primary endpoint, while the long-term infected subjects in the community were assigned to the secondary endpoint. The primary cohort consisted of 107 newly catheterized subjects whose bladders were not infected (Table 1). The secondary cohort consisted of 81 long-term catheter users whose bladders were infected but had no clear evidence of infection (asymptomatic); yet symptoms such as fever requiring antibiotic treatment (symptomatic) are community-based subjects.

In the NHS hospital cohort, the primary end points including time to infection, number of days of infection, and incidence of infection were evaluated.

### 2.1. Time to Infection

**Figure 1:**
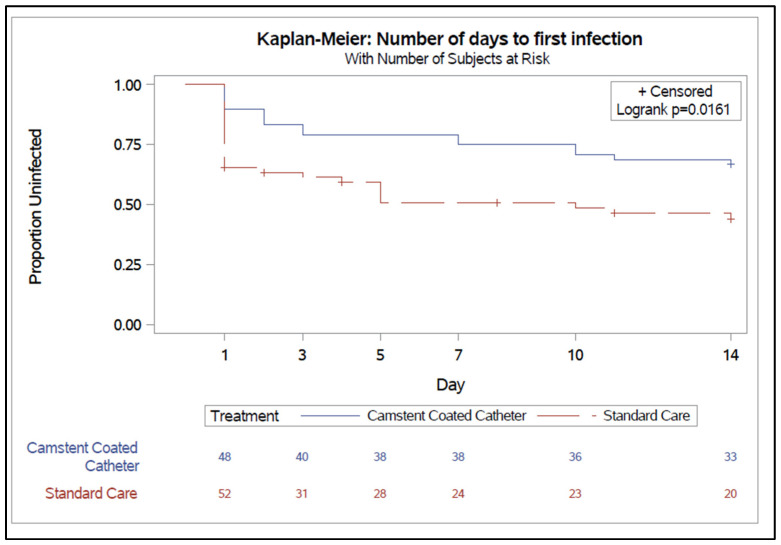
Time to develop symptomatic CAUTI.

A greater proportion of participants in the CCC group remained infection-free compared with the Standard Care (SC) group at day 7 (38 subjects [79%] vs. 24 subjects [46%]) and day 14 (33 subjects [69%] vs. 20 subjects [39%]), respectively (Figure 1). Time-to-event analysis demonstrated a statistically significant difference in time to first infection between groups, as assessed by the log-rank test (*p* = 0.016). This shows that infections occurred earlier and more frequently in the SC group, whereas participants in the CCC group experienced a longer duration without infection.

### 2.2. Number of Days of Infection

**Figure 2:**
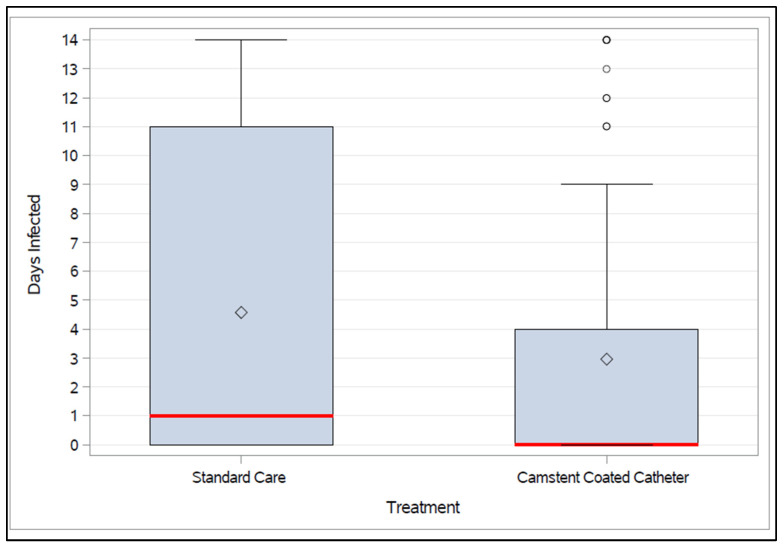
Median (red line), mean (diamond) and interquartile range of the number of days infected in the initially uninfected hospital cohort. Lower outliers are <Q1 − 1.5 × IQR; Upper outliers are >Q3 + 1.5 × IQR.

The mean number of days infected during the first 14 days was 4.6 and 3 days in subjects in SC and CCC groups respectively (Figure 2) with a statistically significant reduction in favor of CCC (two-sided; *p* = 0.0117). The CCC group have fewer days infected, indicating that the catheter coating reduced infection duration compared with the SC.

### 2.3. Incidence of Infection

**Figure 3:**
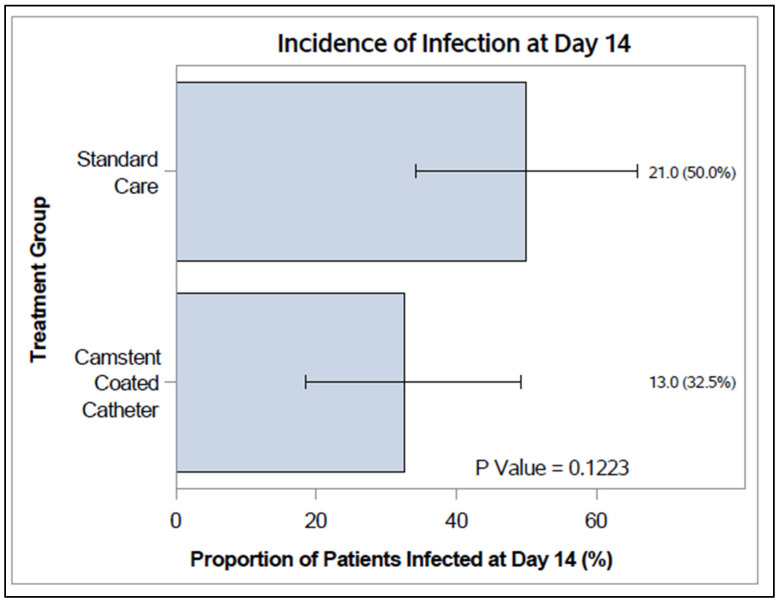
Incidence of infection (%) on Day 14, with 95% confidence interval. *p*-values from Fisher’s exact test. For PP. Footnote: n (%) = number (and proportion) of infected participants.

The incidence of infection was lower in CCC compared with the SC group at day 14 (33% versus 50%) and consistently lower at days 21 and 28 (Figure 3) but not statistically significant (*p* > 0.05).

In the initially infected community care group, the secondary end point and time to symptoms were evaluated in secondary cohorts and UTI antibiotic use whereas PROs and catheter blockage were evaluated for the whole PP group.

### 2.4. Time to Symptoms

**Figure 4:**
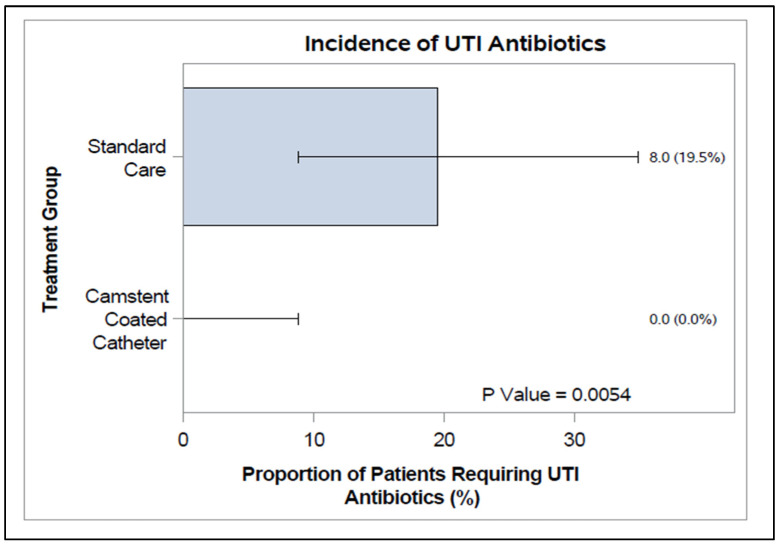
Incidence of UTI antibiotic usage (%), with 95% confidence interval, for the PP population (secondary endpoint subgroup). *p*-values from Fisher’s exact test.

The incidence of symptomatic and asymptomatic UTI cases in the secondary cohorts who were community-based subjects was defined as the first use of antibiotics for symptomatic UTI. No participants in the CCC group developed symptomatic UTIs (0/40 subjects) compared with 20% (8/41 subjects) in the SC group (Figure 4). This difference was statistically significant (*p* = 0.0054). The median number of days to symptoms in the SC group was 9.0 days.

### 2.5. UTI Antibiotic Use

**Figure 5:**
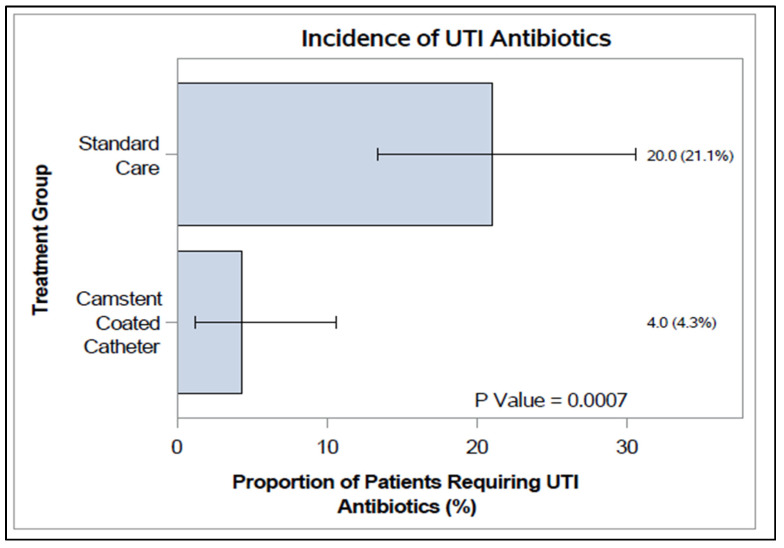
Incidence of patient’s requiring antibiotics for a UTI (%), with 95% confidence interval, for the PP population (all participants). *p*-values from Fisher’s exact test.

Expanding this UTI incidence comparison to include subjects in both the hospital and community-based cohorts, the symptomatic cases were found to be 4.3% (4/93 subjects) and 21.1% (20/95 subjects) and median time to symptoms was 13.5 and 7 days in the CCC and control SC groups respectively (Figure 5) using Fisher’s exact test for statistical significance. There were 21.1% and 4.3% subjects requiring antibiotics in the SC versus the CCC group (absolute reduction 16.8%; relative reduction ~80%). There was a statistically significant difference (*p* = 0.0007) in the usage of UTI antibiotics.

### 2.6. Dipstick Results and Blockages

The Dipstick test results showed no significant results. In standard practice, a DDipstick test would be carried out before a urine sample is sent for CSU. In this study a CSU was carried out at every visit regardless, please see Supplementary Information. The use of dipstick test strips for screening UTIs was associated with a high rate of false positive and false negative results when compared to the gold standard culture method [41,42]. Therefore, the Dipstick nitrite test alone should not be relied upon as the sole method for UTI screening.

In total, in the SC and CCC groups catheter blockage was observed in one subject (1%) and 13 subjects (13.1%) respectively, resulting in early withdrawal from the study. Out of 14 catheter blockage subjects, 9 patients were from spinal injuries.

### 2.7. Catheter Insertion and Removal

During catheter insertion, subject discomfort was observed; the CCC group (22 subjects) showed no discomfort (n = 13; 59.2%), mild discomfort (n = 7; 31.8%), moderate discomfort (n = 2; 9.1%) and no severe discomfort; the SC group (30 subjects) showed no discomfort (n = 22; 73.3%), mild discomfort (n = 5; 16.7%), moderate discomfort (n = 2; 6.7%) and no severe discomfort (n = 1; 3.3%).

During catheter removal, subject discomfort was observed; the CCC group (22 subjects) showed no discomfort (n = 17; 77.3%), mild discomfort (n = 4; 18.2%), moderate discomfort (n = 1; 4.5%) and no severe discomfort; the SC group (30 subjects) showed no discomfort (n = 22; 73.3%), mild discomfort (n = 6; 20.0%), moderate discomfort (n = 1; 3.3%) and no severe discomfort (n = 1; 3.3%).

### 2.8. Safety

Overall, 74 subjects (37.0%) reported adverse events and of these 68 subjects (34.0%) experienced Treatment Emergent Adverse Events (TEAEs). Of those that had a TEAE, 32 subjects (32.3%) were in the CCC group and 36 subjects (35.6%) in the SC group. Although most TEAEs were mild (n/N observed 51/200, 25.5%) or moderate in intensity (n/N observed 16/200, 8.0%), there were also two severe events and two SAEs, with one subject in each group for both categories. There were no life-threatening events or deaths. Approximately half the TEAEs (50/88, 57%) were considered by the Investigator to not be related or unlikely to be related to the intervention. The study intervention was discontinued in 6 subjects (approx. 6.0%) in both groups, and medical intervention was required in 16 (15.8%) subjects in the control SC group compared with only 8 (8.1%) in the CCC group. Furthermore, one subject in the SC group required hospitalization.

## 3. Discussion

This multi-center, randomized study demonstrated CCCs provide clinically meaningful benefits over standard uncoated catheters particularly with respect to significant reductions in both CAUTIs and in time to infection (*p* = 0.016). The reduction in the number of infected days among participants using CCC shows that, in addition to delaying the onset of infection, the catheter coating reduces the overall duration of infectious episodes. Shorter infection duration has clinically relevant implications, including reduced symptom burden, decreased need for antimicrobial therapy, and potential reductions in healthcare resource utilization when compared with Standard Care.

The Camstent catheter in hospitalized patients (primary cohorts) reduced the number of infected days during the first 14 days, with subjects experiencing significantly fewer days of infection compared with SC (*p* = 0.0117).

As samples were collected at discrete time points (Day 14, Day 21, and Day 28), this may indicate the need to assess colonization longitudinally over a defined time period rather than at single sampling days. Such an approach may require a larger sample size to demonstrate statistical significance or to better characterize whether the coating’s effectiveness increases with longer in situ duration. However, the directional consistency across all timepoints supports a beneficial effect of the coating.

Secondary outcomes further highlight the clinical relevance of this reduction in early colonization. No symptomatic cases occurred in the CCC group within the secondary cohort, compared with 19.5% in the SC group (*p* = 0.0054). In the broader PP population, UTI-related antibiotic usage was markedly lower with the coated catheter, with an approximate 80% relative reduction (*p* = 0.0007). This large reduction also helps limit unnecessary antibiotic use and supports better patient care. The absence of symptomatic UTI in the CCC group within the community-based cohort suggests a clinically meaningful protective effect, particularly in real-world settings where prolonged catheterization and delayed recognition of infection are common. The earlier onset of symptoms observed in the SC group further supports the role of catheter coating in delaying or preventing progression from colonization to symptomatic infection. These findings are of particular relevance in community care, where reductions in symptomatic infection may translate into decreased antibiotic prescribing, fewer unplanned healthcare contacts, and improved patient outcomes.

In the SC and CCC groups, the catheters were blocked in one subject (1%) and 13 subjects (13.1%) respectively resulting in early withdrawal from the study.

Factors such as complex comorbidities, recurrent urinary tract infections, chronically colonized bladders, prolonged bed rest, impaired mobility compromising posture, catheter positioning, reducing consistent flow, and inadequate hydration, compounded by paralysis and limited hand function further concentrates urine leads to higher rates of catheter blockages.

In a first-in-man prospective pilot study Kalenderski et al., analyzed biofilm biomass and biomineralization using uropathogens *Proteus mirabilis* and *Pseudomonas aeruginosa* on uncoated (silicone) and coated catheters from hospitalized subjects which were quantified by confocal microscopy using fluorescently tagged bacteria or stained for biofilm and mineral. In a total of 161 patients, there was no blockage observed in the CCC group, and large crystals were identified in silicone catheter group. The mineral deposition was very different in the CCC group from that observed in the silicone catheter group. The study resulted in significantly lower median biofilm biomass and mineral deposition in the CCC group with no blockages reported, consistent with the lack of encrustation observed in the in vitro bladder model [36].

Future research should prioritize health-economic analyses to assess potential reductions in infection-related admissions, antibiotic use, nursing time, and overall catheter-associated care costs. Demonstrating meaningful cost savings alongside clinical benefits will be essential in supporting widespread adoption within healthcare systems. Additional studies could include direct comparisons between CCC and other coated catheter technologies, as well as focused evaluation within specific patient subgroups, such as long-term catheter users and patients at higher risk of catheter-associated infection.

Overall, the results consistently show that the CCC delays infection, shortens infection duration, reduces symptomatic presentations, and significantly lowers usage of UTI antibiotics. The findings also demonstrate that the Camstent M4D coating reduces surface friction, which prevents biofilm formation and enhances patient comfort during insertion and withdrawal [36]. In addition, the advantages of coated catheters may help avoid the costs associated with treating catheter-related infections and reduce the spread of antibiotic-resistant pathogens. While larger randomized trials are needed to confirm these findings and define long-term outcomes, the current evidence suggests that the CCC provides a meaningful advantage over standard catheters in mitigating catheter-associated infections.

## 4. Methods

### 4.1. Subjects

Uncoated silicone catheter (SC) and Camstent Coated Catheters (CCC) were used in this study. The Camstent Coated Catheters are manufactured by Camstent Ltd., Bedford, UK. Adults undergoing urethral catheterization with an anticipated duration of between 7 and 28 days were identified at UK participating sites. Study inclusion criteria were as follows: (i) Adults aged > 18 years requiring the insertion or exchange of a Foley catheter as a component of their routine clinical care as per guidelines, (ii) Subjects that understand and are willing to participate in the study and are able to comply with study procedures and visits. The North of Scotland Research Ethics Committee and Health Research Authority approved the study protocol, and each subject gave written informed consent.

Subjects were excluded (i) if they recently (within 3 weeks) had a urinary catheter and displayed symptoms of current urinary tract infection), (ii) were pregnant or breastfeeding, (iii) had a potentially immunocompromised condition, (iv) had a known silicone allergy or sensitivity, with asymptomatic infection (if required may need a blood test for confirmation), (v) had a known bloodstream infection or an infection that requires prolonged antibiotic therapy, (vi) were participating in any other clinical study, (vii) used an investigational drug or device within four weeks prior to study entry that may interfere with this study, (viii) were on any medication deemed by the Investigator to potentially interfere with the study treatment.

### 4.2. Study Design and Procedure

**Figure 6:**
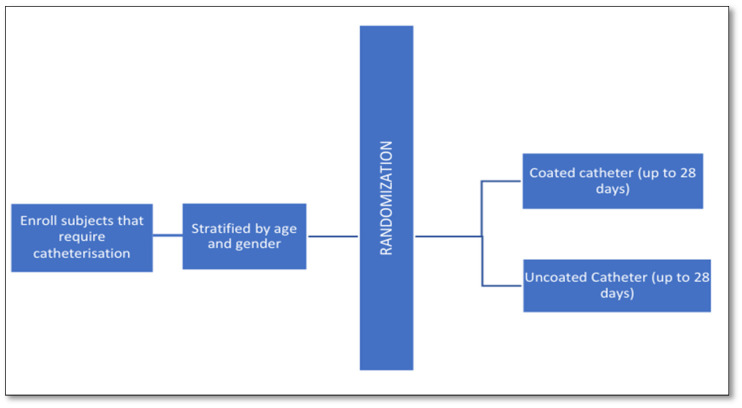
Schematic picture of the study flow.

This was a prospective, multi-center, randomized study conducted in the UK for a period of 5 years. A schematic picture of the study flow is provided below in Figure 6.

Randomization was 1:1 using the Castor EDC platform with variable block size. Patients were stratified by age and gender. During the first visit, urine samples were obtained, and each subject’s temperature was recorded. During the screening phase, trial participants signed an informed consent form (ICF). During the treatment phase, eligible participants were randomly assigned to either a coated or uncoated IUC (1:1). The subjects were assessed on days 3, 5, 7, 10, 14, 21, and 28 until the catheter was removed, or if the subject required antibiotics. Once the catheter was removed the subject would be removed from the trial and receive their routine catheter. Urine testing was included in the efficacy evaluations at each visit and urine samples were cultured on agar plates to determine the CFU/mL for each urine sample. All randomized participants were followed for up to 28 days from the time of catheterization, or until the individual withdrew or was discharged from the hospital, whichever occurred first. Any subjects who did not complete the trial, prematurely discontinued, or were recognized as lost-to-follow-up are documented and recorded. The study trial procedures carried out on each visit are summarized in Table 2.

The study was conducted in accordance with the specifications of the protocol (Cam-Cath-001) and in accordance with principles consistent to the declaration of Helsinki (6th revision 2008), ISO 14155 [43], Good Clinical Practice (GCP) and currently applicable regulations. The study device used in this study was manufactured, handled, and stored in compliance with Good Manufacturing Practices (GMP), and was only used as per the protocol.

### 4.3. Endpoints

The primary efficacy endpoint was a reduction in the number of days with bacterial viable counts > 10^5^ CFU/mL in the CCC compared with the SC. The secondary efficacy endpoint was patient reported outcomes (PROs), reduction in symptomatic bacteriuria (CAUTI) with coated catheters versus uncoated controls, reduction in prophylactic or therapeutic antibiotic use during catheterization, and reduction in catheter blockages. Sample size was based upon a 10% relative reduction in the intervention group.

The primary cohort included 107 newly catheterized individuals with no evidence of bladder infection. The secondary cohort comprised 81 long-term catheter users who had bladder infections without clear clinical signs (asymptomatic), as well as cases presenting symptoms such as fever requiring antibiotic treatment (symptomatic); all participants were community-based.

### 4.4. Statistical Analysis

Sample Size Determination and Rationale: The sample size calculation is based on pilot data and gives an estimate of effect size. Using the 14-day measure as the primary endpoint, with the expectation of 50% of individuals in control being above the threshold at that time point. The sample size was based upon a 10% relative reduction in the intervention group. Aiming for 90% statistical power, the planned total sample size of 258 (129 in each group) with analyzable data at 14 days. With an allowance for a 5% drop-out of the total 272.

Analysis Populations: The Intent-to-Treat population was defined as the set of randomized participants who had at least one post randomization efficacy assessment for the reduction in number of days of bacteriuria. The ITT population was the primary population for the analysis of primary and secondary endpoints. The Per Protocol population was defined as the set of participants who met the ITT population requirements, defined above, and who were not associated with a major protocol violation/deviation. This population was identified before the database lock. The safety population was defined as the set of participants receiving treatment after randomization. This population was used for the analysis of safety parameters.

Analysis Methods: A Statistical Analysis Plan (SAP) was developed and approved before the database was locked. The SAP presents the detailed statistical methodology used in analyzing the efficacy and safety data from this study.

## 5. Conclusions

Camstent Coated Catheters (CCCs) demonstrated substantially higher infection-free rates than standard care (SC), with 79% versus 46% at Day 7 and 69% versus 39% at Day 14. Notably, within the secondary cohort of long-term catheterized patients, no symptomatic catheter-associated urinary tract infections (CAUTIs) were observed in the CCC group, whereas the incidence was 20% in the standard care (SC) group. In addition, antibiotic use for urinary tract infections was markedly lower among patients receiving CCCs (4.3%) relative to those receiving standard care (21.1%). These findings support the adoption of CCCs as part of strategies to prevent CAUTIs and promote antimicrobial stewardship, particularly in the context of the ongoing limitations of traditional prevention measures such as antibiotic prophylaxis and standard catheter care practices, which have not fully mitigated infection risk. The observed benefits highlight the value and clinical need for novel catheter technologies such as CCC.

## Figures and Tables

**Table 1:** Primary Cohort subjects in PP populations in NHS hospitals.

	Standard Care Catheter (SC)	Camstent Coated Catheter (CCC)
ITT Population	101	99
ITT—Primary Endpoint Subgroup	57	57
ITT—Secondary Endpoint Subgroup	44	42
PP Population	95	93
PP—Primary Endpoint Subgroup	54	53
PP—Secondary Endpoint Subgroup	41	40

**Table 2:** Trial Procedure at each visit.

Clinical Study Schedule										
Time Cycle	Visit-1 Screening	Visit-2 Post-Catheterization	Visit-3 Day 3	Visit-4 Day 5	Visit-5 Day 7	Visit-6 Day 10	Visit-7 Day 14	Visit-8 Day 21	Visit-9 Day 28	Removal of Catheter
Informed Consent	x									
Inclusion Exclusion Criteria	x									
Demographic and Medical History	x									
Concomitant Medication Review	x	x	x	x	x	x	x	x	x	x
Review Adverse Events	x	x	x	x	x	x	x	x	x	x
Vital Signs	x									
Randomization	x									
Temperature (a)	x	x	x	x	x	x	x	x	x	x
Mid-Stream Urine Sample	x									
Urine Dipstick Test	x	x	x	x	x	x	x	x	x	x
Catheter Urine Sample	x	x	x	x	x	x	x	x	x	x
Urinalysis (b)	x	x	x	x	x	x	x	x	x	x
Patient Reported Outcomes (c)	x	x	x	x	x	x	x	x	x	x
Nurse Reported Outcomes	x	x	x	x	x	x	x	x	x	x
Catheter Analysis (d)			x	x	x	x	x	x	x	
Informed Consent	x									
Inclusion Exclusion Criteria	x									
Demographics and Medical History	x									
Concomitant Medication Review	x	x	x	x	x	x	x	x	x	x
Review Adverse Events	x	x	x	x	x	x	x	x	x	x
Vital Signs	x									
Randomization	x									
Temperature (a)	x	x	x	x	x	x	x	x	x	x
Mid-Stream Urine Sample	x									
Catheterization	x									
Urine Dipstick Test	x	x	x	x	x	x	x	x	x	x
Catheter Urine Sample	x	x	x	x	x	x	x	x	x	x
Urinalysis (b)	x	x	x	x	x	x	x	x	x	x
Patient Reported Outcomes (c)	x	x	x	x	x	x	x	x	x	x
Nurse Reported Outcomes	x	x	x	x	x	x	x	x	x	x
Catheter Analysis (d)			x	x	x	x	x	x	x	

Footnotes: (a) BodyTemperature to be taken. (b) Urine samples to be sent to laboratories within 24 h of sample taken. (c) EuroQol EQ-5D-5L to be completed before any study procedures. (d) First 10 blocked catheters (5 SC and 5 CCC) to be sent to the laboratory. Where (x) is listed procedures need to be carried out.

## Data Availability

Data is available upon request.

## Supplementary Materials

The following supporting information can be downloaded at: https://www.mdpi.com/article/10.3390/antibiotics15040369/s1, Supplementary Information: Antibiotics prescribed for a UTI and Urinalysis results. Table S1: Antibiotics prescribed. Table S2: Urinalysis Results.

## Abbreviations

The following abbreviations are used in this manuscript:

CAUTIs	Catheter-associated urinary tract infections
CCC	Camstent Coated Catheters
CFU/mL	Colony-forming units per milliliter
GCP	Good Clinical Practice
GMP	Good Manufacturing Practice
IUCs	Indwelling urinary catheters
ICF	Informed consent form
ITT	Intention to Treat
NHS	National Health Service
NMA	Noble metal alloys
PP	Per Protocol
PRO	Patient-reported outcomes
SAP	Statistical Analysis Plan
SC	Standard Care
UTIs	Urinary tract infections
US	United States
