# Supplementary material for: A Prospective Multicenter Randomized Study to Assess the Impact of a Novel Catheter Coating on Clinical Bacteriuria
Source: Antibiotics (Basel). 2026 Apr 3;15(4):369. doi: 10.3390/antibiotics15040369 (PMC13113331; doi:10.3390/antibiotics15040369)
Supplement: Supplementary file 1 [file antibiotics-15-00369-s001.zip › antibiotics-4204065-supplementary.pdf]

Supplementary Information
for
A Prospective Multicentre Randomized Study to Assess the Impact of a Novel Catheter Coating on Clinical Bacteriuria

Mark Rochester [1], *, Catherine Rennie [1], Clare Hayes [1], Jean O'Driscoll [2] and Maurizio Belci [2]
[1] **Norfolk & Norwich University Hospital and Norwich UK**
[2] **Stoke Mandeville Hospital, Aylesbury, UK**
* **Correspondence: Author, Mark.Rochester@nnuh.nhs.uk**

Table S1. Antibiotics prescribed

Below is a list of medication patient's were prescribed for UTI's

Nitrofurantoin
Tazocin
Gentamycin
Trimethoprim
Amoxicillin
Nitrofurantoin
Pivmecillinam
Meropenem
Furantoin
Co-amoxiclav
Piperacillin/Tazobactam
Methanamine Hippurate

Table S2. Urinalysis Results

Below is a list of bacteria from the urinalysis results

**Bacterial Species**	**Number of patients**
Mixed growth	45
Escherichia coli	31
Proteus	10
Klebsiella	22
Pseudomonas Aeruginosa	17
Enterobacter Cloacae	8
Citrobacter Koseri	8
Enterococci	14
Candida	4
Coliform	8
Staphylococcus	10
Morganella morganii	2
Pseudomonas putida	1
Serratia marcescens	1
Saccharomyces cerevisiae	1
